# Supercurrent switch in *π* topological junctions based upon a narrow quantum spin Hall insulator

**DOI:** 10.1038/s41598-017-10960-4

**Published:** 2017-09-06

**Authors:** Qingyun Yu, Ze Tao, Juntao Song, Y. C. Tao, Jun Wang

**Affiliations:** 1 Department of Physics and Institute of Theoretical Physics, Nanjing Normal University, Nanjing, 210023 China; 2 College of Telecommunications & Information Engineering, Nanjing University of Posts and Telecommunications, Nanjing, 210023 China; 3 Department of Physics and Hebei Advanced Thin Film Laboratory, Hebei Normal University, Shijiazhuang, 050024 China; 4 Department of Physics, Southeast University, Nanjing, 210096 China

## Abstract

The narrow quantum spin Hall (QSH) insulator is characterized by interedge coupling, which could feature exotic transport phenomena, and thus serves as the key element for topological superconducting electronic devices. Herein, we theoretically explore possible Josephson *π* states in a QSH insulator strip touching on two *s*-wave superconductors in the presence of the interedge coupling. It is shown that the interedge coupling could give rise to a 0 − *π* transition modulated by the gate voltage, originating from an additional *π* phase difference caused by the interedge backscattering. The 0 − *π* transition in turn can manifest the helical spin texture of the edge states. A considerable residual value of the supercurrent at the 0 − *π* transition point is always exhibited, suggesting a very efficient performance of the device as a supercurrent switch. Moreover, the region of coexisting 0 and *π* states is found fairly large, which can be used to improve accuracy in the design of a *π* superconducting quantum interference device.

## Introduction

The quantum spin Hall (QSH) insulator, a kind of two-dimensional topological insulator, is a topologically nontrivial phase of electronic matter^1–4^. The transport of the QSH insulator is characterized by gapless helical edge states, which are protected by the time reversal symmetry. The spin-up electrons propagate clockwise along the sample edge, while the spin-down do counterclockwise, indicating that the intraedge backscattering is prevented^5^. Therefore, the helical edge states have not only the physical significance but also important applications in topological superconducting spintronics and topological quantum computation^1–4^. Besides, while the edge-state conduction^3, 4^ and the spin polarization of the edge current^6^ have been verified, a direct evidence for the helical spin texture of the edge states remains a challenge^7^. For a narrow QSH insulator with two edges getting close to each other, the overlap between edge states from opposite edges produces an energy gap, leading to the so-called interedge coupling, although the intraedge backscattering is still forbidden^8^. Resultantly, the properties of QSH insulator can be greatly modified and one remarkable property is the elastic interedge backscattering between the edge states at the two sides^7–9^.

Although a variety of Josephson junctions, on the other hand, have been proposed and observed, so far, the reports on the ones based on narrow QSH insulators lack enough^10^. One peculiar feature of some Josephson junctions is the 0 − *π* transition^10–14^, the phase difference *ϕ* is usually zero in the ground state, however, an extra *π* phase difference can emerge under appropriate conditions, and so the maximum or critical Josephson current is reversed. The junction with such a *π* phase difference is called a *π* junction, or deemed as a supercurrent switch, which can be used as a basic component in superconducting qubits and quantum computing and information^10–18^. Moreover, the stable and metastable 0 and *π* states in the crossover coexisting region can yield two flux jumps per one external flux quantum in a superconducting quantum interference device (SQUID)^19, 20^, indicating a potential application in quantum electronics^21^. However, most Josephson junctions generating the 0 − *π* transition have their disadvantages. For instance, the one induced by the ferromagnet (FM) exchange energy or the FM layer length of the Josephson junction^22–24^ is not only of difficulty in modulation but also of high-energy in dissipation, being not expected in the quantum qubit application^16–18^. Hence, it is still highly desirable that a Josephson *π* junction owns low-energy dissipation and much convenience in manipulation simultaneously. Due to the helicity conservation of the carriers, the electron travels in the QSH insulator edge dissipationlessly^3–5^, which can be therefore a good candidate for the *π* junction. Particularly, it is expected to maximize the potential for transport phenomena induced by the interedge backscattering in topological superconducting electronics based on a narrow QSH insulator.

**Figure 1:**
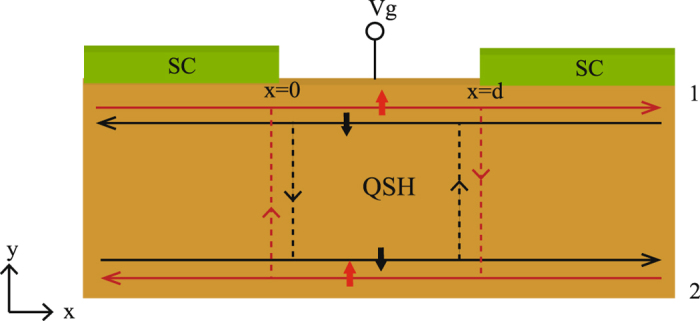
Schematic illustration of the proposed Josephson junction with a gate voltage *V*_*g*_ applied on the middle normal segment of width *d*. The QSH insulator strip with two edges (edges 1 and 2) is sketched as the orange bar. The red and black lines illustrate the helical edge states with the vertical arrows representing the spin orientation, and the interedge coupling causes interedge backscattering indicated by the dotted lines. The two green bars denote the two *s*-wave SCs deposited on edge 1.

In this letter, we therefore propose a Josephson junction fabricated on the narrow QSH insulator strip with the longitudinal direction along the *x* axis as shown in Fig. 1, where two *s*-wave superconductors (SCs) are in intimate contact with one edge of the strip (edge 1)^25^ and the middle normal segment sandwiched between them is applied by a gate voltage *V*_*g*_. Compared with the interedge coupling strength *α*(*x*) in the middle region (0 < *x* < *d*) assumed to be *α*_2_, a lower one *α*_1_ exhibits in the left and right regions, stemming from that the wave functions of electrons in the QSH insulator strip can penetrate into the bulk SC^26^. Due to the proximity effect, a superconducting pair potential is induced in the contacting areas^25, 27^, and its amplitude depends upon the coupling between the edge and the SC. Only edge 1 in touch with the SC can be assumed superconducting while edge 2 keeps normal thanks to the superconducting gap penetrating into the QSH insulator with only a few atomic layers^28^. In the proposed setup, owing to the interedge backscattering, a 0 − *π* transition is found to be manipulated by the gate voltage *V*_*g*_, which embodies the helical spin texture of the edge states in the QSH insulator. It is also shown that there exists not only a fairly large residual value of critical Josephson current at the 0 − *π* transition point but also a large region of coexisting 0 and *π* states. The results pave the way toward the designs of a low-energy dissipation supercurrent switch with high efficiency and a *π* SQUID with improved accuracy.

### Topological Josephson junctions with interedge coupling

Due to the edge states in the QSH insulator strip protected by the time reversal symmetry, no spin-flip effects are assumed^29^. Hence, we can apply the four-spinor wave function ${{\rm{\Psi }}}_{\sigma }\,(x)={({u}_{1\sigma }(x),{u}_{2\sigma }(x),{v}_{1\bar{\sigma }}(x),{v}_{2\bar{\sigma }}(x))}^{T}$ for this system, where *σ* denotes the spin orientation (*σ* = ↑, ↓), $\bar{\sigma }$ is opposite to *σ*, the wave functions *u*_1(2)_ and *v*_1(2)_ stand for the probability amplitudes of the electron and hole in edge 1(2), respectively. The Bogoliubov-de Gennes (BdG) equation^30^ is given by

1 $${{\rm{H}}}_{BdG}{{\rm{\Psi }}}_{\sigma }\,(x)=E{{\rm{\Psi }}}_{\sigma }\,(x)$$

with *E* the quasipartical energy measured from the Fermi energy *E*_*F*_ and ref. 29

$${H}_{BdG}=(\begin{array}{cccc}{h}_{1\sigma } & \alpha \,(x) & {\rm{\Delta }}\,(x) & 0\\ \alpha \,(x) & {h}_{2\sigma } & 0 & 0\\ {{\rm{\Delta }}}^{\ast }\,(x) & 0 & -{h}_{1\sigma } & -\alpha \,(x)\\ 0 & 0 & -\alpha \,(x) & -{h}_{2\sigma }\end{array}),$$

where ${h}_{\mathrm{1(2)}\sigma }=\mp i\hslash {\upsilon }_{F}{\partial }_{x}-\mu \,(x)$ is the Dirac-like Hamiltonian for helical particles in the two edges with the Fermi velocity *υ*_*F*_, the chemical potential *μ*(*x*), and the positive (negative) sign for spin-down (spin-up) electrons in edge 1 and spin-up (spin-down) electrons in edge 2. *μ*(*x*) is assumed 0 for the two superconducting regions and is controlled by a gate voltage *V*_*g*_ for the middle region. The superconducting pair potential ${\rm{\Delta }}(x)={\rm{\Delta }}{e}^{i{\varphi }_{L(R)}}$ for the left (right) superconducting region exists only in edge 1 with *ϕ*_*L*(*R*)_ the superconducting phase, while Δ(*x*) = 0 for the middle. The temperature dependence of Δ is given by ${\rm{\Delta }}\equiv {\rm{\Delta }}\,(T)={{\rm{\Delta }}}_{0}\,\tanh \,(1.74\sqrt{{T}_{c}/T-1})$. The macroscopic phase difference across the junction is defined as *ϕ* = *ϕ*_*R*_ − *ϕ*_*L*_.

For the injection of an electron-like quasipartical (ELQ) with energy *E* > Δ from the left SC region, with the general solution of Eq. (1), the wave function in the left *s*-wave SC region is given by

2 $${{\rm{\Psi }}}_{L}\,(x)={\psi }_{L+}^{e}+{b}_{1}{\psi }_{L-}^{e}+{a}_{1}{\psi }_{L-}^{h}$$

for *x* < 0, where ${\psi }_{L\pm }^{e}$ = $\{{\eta }^{\pm }(E\pm \hslash {\upsilon }_{F}{k}^{e})/{\alpha }_{1}$, ${\eta }^{\pm }$, ${\eta }^{\pm }\lambda {e}^{-i{\varphi }_{L}}/({\alpha }_{1}\,{\rm{\Delta }})$, ${-{\eta }^{\pm }\lambda {e}^{-i{\varphi }_{L}}/[(E\mp \hslash {\upsilon }_{F}{k}^{e}){\rm{\Delta }}]\}}^{T}{e}^{\pm i{k}^{e}x}$ with $\lambda \,=$ ${E}^{2}-{(\hslash {\upsilon }_{F}{k}^{e})}^{2}-{\alpha }_{1}^{2}$ and ${\eta }^{\pm }$ = $\sqrt{{|(E\pm \hslash {\upsilon }_{F}{k}^{e})/{\alpha }_{1}|}^{2}+1+{|\lambda /({\alpha }_{1}{\rm{\Delta }})|}^{2}+{|\lambda /[(E\mp \hslash {\upsilon }_{F}{k}^{e}){\rm{\Delta }}]|}^{2}}$, and ${\psi }_{L\pm }^{h}$ = $\{{\gamma }^{\pm }(E\pm \hslash {\upsilon }_{F}{k}^{h})/{\alpha }_{1}$, ${\gamma }^{\pm }$, ${\gamma }^{\pm }\nu {e}^{-i{\varphi }_{L}}/({\alpha }_{1}\,{\rm{\Delta }})$, ${-{\gamma }^{\pm }\nu {e}^{-i{\varphi }_{L}}/[(E\mp \hslash {\upsilon }_{F}{k}^{h}){\rm{\Delta }}]\}}^{T}{e}^{\pm i{k}^{h}x}$ with ${\gamma }^{\pm }$ = $\sqrt{{|(E\pm \hslash {\upsilon }_{F}{k}^{h})/{\alpha }_{1}|}^{2}+}$ $\sqrt{1+{|\nu /({\alpha }_{1}{\rm{\Delta }})|}^{2}+{|\nu /[(E\mp \hslash {\upsilon }_{F}{k}^{h}){\rm{\Delta }}]|}^{2}}$ and $\nu ={E}^{2}-{(\hslash {\upsilon }_{F}{k}^{h})}^{2}-{\alpha }_{1}^{2}$. The wave vectors in the SC regions are given by ${k}^{e(h)}$ = $\sqrt{{E}^{2}-{\alpha }_{1}^{2}-\frac{1}{2}{\rm{\Delta }}\,({\rm{\Delta }}+(-)\sqrt{{{\rm{\Delta }}}^{2}+4{\alpha }_{1}^{2}})}/(\hslash {\upsilon }_{F})$. The coefficients *a*_1_ and *b*_1_ are, respectively, the amplitudes of the Andreev reflection (AR) as a hole-like quasipartical (HLQ), and normal reflection as an ELQ. In the middle region, we have the wave function

3 $${{\rm{\Psi }}}_{M}\,(x)={g}_{1}{\psi }_{1}+{g}_{2}{\psi }_{2}+{g}_{3}{\psi }_{3}+{g}_{4}{\psi }_{4}$$

for 0 < *x* < *d*, where ${\psi }_{1}$ = ${[\cos (\theta /2),\sin (\theta /2),0,0]}^{T}{e}^{i{q}^{e}x}$ with sin *θ* = *α*_2_/(*E* − *V*_*g*_), ${\psi }_{2}[\,\sin \,(\theta /2),$ = ${\cos (\theta /2),0,0]}^{T}{e}^{-i{q}^{e}x}$, ${\psi }_{3}$ = ${[0,0,-\sin (\phi /2),\cos (\phi /2)]}^{T}{e}^{i{q}^{h}x}$ with sin *φ* = *α*_2_/(*E* + *V*_*g*_), and ${\psi }_{4}$ = ${\mathrm{[0},0,-\cos (\phi /2),\sin (\phi /2)]}^{T}{e}^{-i{q}^{h}x}$. The wave vectors in the middle region are given by ${q}^{e(h)}$ = $\sqrt{{(E-(+){V}_{g})}^{2}-{\alpha }_{2}^{2}}/(\hslash {\upsilon }_{F})$. Amplitudes of electrons and holes propagating in the middle region are given by the coefficients *g*_*i*_(*i* = 1 − 4). The wave function in the right *s*-wave SC region is given by

4 $${{\rm{\Psi }}}_{R}\,(x)={c}_{1}{\psi }_{R+}^{e}+{d}_{1}{\psi }_{R+}^{h}$$

for *x* > *d*, where coefficients *c*_1_ and *d*_1_ are, respectively, the amplitudes of the transmission to the right SC as an ELQ, and transmission to the right SC as a HLQ. ${\psi }_{R\pm }^{e}$ and ${\psi }_{R\pm }^{h}$ can be respectively obtained from ${\psi }_{L\pm }^{e}$ and ${\psi }_{L\pm }^{h}$ by making an exchange between *L* and *R*.

All the coefficients *a*_1_, *b*_1_, *c*_1_, *d*_1_, and *g*_*i*_ will be determined by matching the boundary conditions

5 $${{{\rm{\Psi }}}_{L}(x)|}_{x={0}_{-}}={{{\rm{\Psi }}}_{M}(x)|}_{x={0}_{+}},\,{{{\rm{\Psi }}}_{M}(x)|}_{x={d}_{-}}={{{\rm{\Psi }}}_{R}(x)|}_{x={d}_{+}}.$$

Analogously, one can easily obtain the AR amplitude *a*_2_ for the injection of a HLQ with energy *E* > Δ from the left SC region. The analytical expressions for *a*_1_ and *a*_2_, are respectively given by

6 $${a}_{1}={A}_{1}+({A}_{2}{e}^{i\varphi }+{A}_{3}{e}^{i{\zeta }^{+}}+{A}_{4}{e}^{-i{\zeta }^{+}}+{A}_{5}{e}^{i{\zeta }^{-}}+{A}_{6}{e}^{-i{\zeta }^{-}})/G$$

and

7 $${a}_{2}={B}_{1}-({B}_{2}{e}^{i\varphi }+{B}_{3}{e}^{i{\zeta }^{+}}+{B}_{4}{e}^{-i{\zeta }^{+}}+{B}_{5}{e}^{i{\zeta }^{-}}+{B}_{6}{e}^{-i{\zeta }^{-}})/G$$

with $G$ = ${C}_{1}{e}^{i\varphi }+{C}_{2}{e}^{-i\varphi }+{C}_{3}{e}^{i{\zeta }^{+}}$ + ${C}_{4}{e}^{-i{\zeta }^{+}}+{C}_{5}{e}^{i{\zeta }^{-}}+{C}_{6}{e}^{-i{\zeta }^{-}}$, where *ζ* ^±^ = *d*(*q* ^*e*^ ± *q* ^*h*^) characterize physically important oscillations of the AR amplitudes *a*_1(2)_, A_*i*_(*i* = 1–6), B_*i*_(*i* = 1–6), and *C*_*i*_(*i* = 1–6) are complex functions of *E*, Δ, *V*_*g*_, *α*_1_, and *α*_2_. Then, the dc Josephson current at a given temperature can be expressed in terms of the AR amplitudes *a*_1_ and *a*_2_ by using the temperature Green’s function formalism^31^

8 $$I\,(\varphi )=\frac{e{\rm{\Delta }}}{4\hslash }{k}_{B}T\,\sum _{{\omega }_{n},\sigma }\,\frac{1}{{{\rm{\Omega }}}_{n}}\,({k}_{n}^{e}+{k}_{n}^{h})\,(\frac{{a}_{1n}}{{k}_{n}^{e}}-\frac{{a}_{2n}}{{k}_{n}^{h}}),$$

where ${k}_{n}^{e},\,{k}_{n}^{h},\,{a}_{1n}$, and *a*_2*n*_ are respectively obtained from *k* ^*e*^, *k* ^*h*^, *a*_1_, and *a*_2_ by the analytic continuation *E* → *iω*_*n*_. The Matsubara frequencies are *ω*_*n*_ = *πk*_*B*_ *T*(2*n* + 1) with *n* = 0, ±1, ±2, …, and ${{\rm{\Omega }}}_{n}=\sqrt{{\omega }_{n}^{2}+{{\rm{\Delta }}}^{2}}$.

The dc Josephson current can be acquired by another formalism^11, 32–34^ $I=\tfrac{2e}{\hslash }\,{\sum }_{i}\tfrac{d{E}_{i}\,(\varphi )}{d\varphi }\,f\,({E}_{i})$ with *f*(*E*_*i*_) the Fermi-Dirac distribution and *E*_*i*_(*ϕ*) = ±*E*(*ϕ*) the two energies for a single pair of Andreev bound states which can be arrived at from the BdG equation. Here, each Andreev bound state carries a current and the ± denotes two possible processes, which differ in direction of propagation of the electrons and holes and correspond to the currents following in opposite directions. In fact, the terms $\tfrac{d{E}_{i}\,(\varphi )}{d\varphi }$ are just corresponding to the ARs terms *a*_1_ and *a*_2_ in Eq. (8), so the two methods are equivalent.

### A 0 − *π* transition induced by interedge backscattering

In the following calculations, the critical or maximum Josephson current, the relevant quantity measured experimentally^22–24^, is defined as *I*_*c*_ = |max{*I*(*ϕ*)}| with the unit of Josephson current^15^ *I*_0_ = 2*e*Δ_0_/*ħ*. We have taken the units *ħυ*_*F*_ = 1 and Δ_0_ = 1 and set the temperature *T* = 0.1*T*_*c*_, where the critical temperature *T*_*c*_ ≈ 0.57Δ_0_/*k*_*B*_ based on the BCS theory.

**Figure 2:**
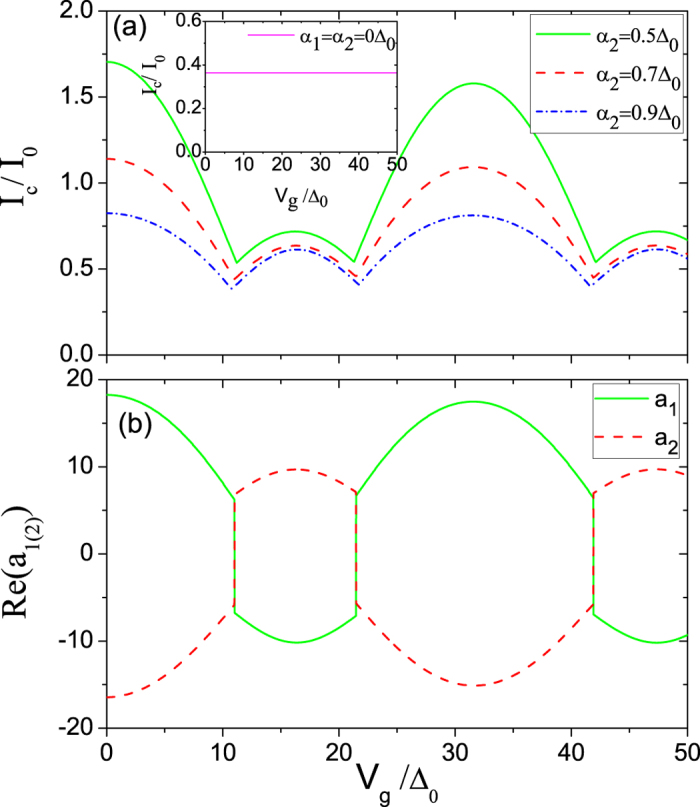
(**a**) The critical Josephson current *I*_*c*_ as a function of gate voltage *V*_*g*_ for different interedge coupling strengths *α*_2_. The inset shows *I*_*c*_ as a function of *V*_*g*_ without interedge coupling. (**b**) Andreev reflection coefficients *a*_1_ and *a*_2_ as a function of *V*_*g*_, corresponding to the red dashed line in Fig. 2(a), coming from the contribution of all channels. Here, *α*_1_ = 0.5*α*_2_, *d* = 0.1*ξ*_0_ with *ξ*_0_ = *ħυ*_*F*_/Δ_0_ superconducting coherence length at zero temperature, and the various *α*_2_ are indicated.

On the basis of Eq. (8), we calculate the critical Josephson current *I*_*c*_ as a function of gate voltage *V*_*g*_ for different interedge coupling strengths *α*_2_, which is illustrated in Fig. 2(a). *I*_*c*_ is shown to oscillate with *V*_*g*_ but decay weakly and has three peaks and three dips. This can be explained by two additional phase factors *ζ* ^±^ in analytical expressions *a*_1(2)_ modulated by *V*_*g*_. The dips in *I*_*c*_ correspond to the 0 − *π* or *π* − 0 transition that can be easily controlled by *V*_*g*_ and thus is very useful for applications. Moreover, the positions of both the first and third dips are found to shift toward the smaller *V*_*g*_ with increasing *α*_2_, however, the position of the second dip toward the bigger *V*_*g*_. In particular, the value for each dip is decreased with the enhancement of *α*_2_, implying that *α*_2_ can suppress the Josephson current. Most interestingly, we find that there are considerable residual values of *I*_*c*_ at the dips, which should be experimentally detectable, as in ref. 15. For comparison, *I*_*c*_ as a function of *V*_*g*_ without interedge coupling is also presented in the inset of Fig. 2(a). With *V*_*g*_ increased, *I*_*c*_ remains constant, indicating no 0 − *π* transition as demonstrated theoretically in refs 34 and 35. This is physically natural as in *s*-wave SC/normal metal/*s*-wave SC structure.

**Figure 3:**
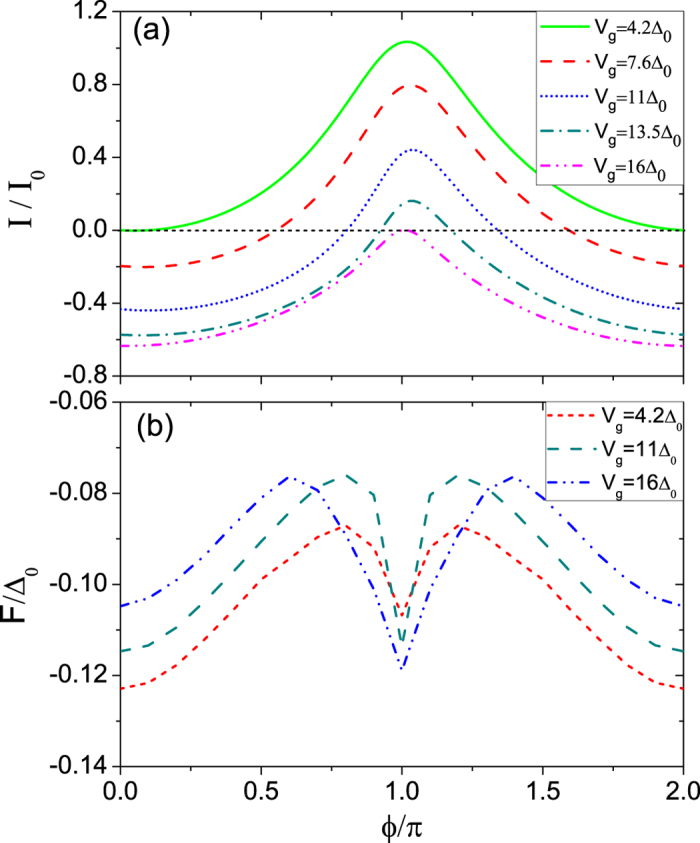
(**a**) Current-phase relations *I*(*ϕ*) with various *V*_*g*_ corresponding to the first dip of red dashed line in Fig. 2(a). Here, the parameters are the same as those as in Fig. 2 except that *α*_2_ = 0.7Δ_0_ and the various *V*_*g*_ are indicated. (**b**) The *ϕ* dependences of the free energy *F* in the junction at the crossover point (*V*_*g*(*c*)_ = 11Δ_0_), 0-state (*V*_*g*_ = 4.2Δ_0_), and *π*-state (*V*_*g*_ = 16Δ_0_).

In order to understand how the AR amplitudes *a*_1(2)_ for the incident ELQ (HLQ) determine the 0 − *π* transition under the interedge coupling, we present in Fig. 2(b), the AR amplitudes *a*_1_ and *a*_2_ as a function of gate voltage *V*_*g*_ at *α*_2_ = 0.7Δ_0_ including the contribution from all channels, corresponding to the red dashed line in Fig. 2(a). With increasing *V*_*g*_, *a*_1_ first decreases from positive value, then abruptly jumps to the negative value at the gate voltage *V*_*g*_ ≡ *V*_*g*(*c*)_ = 11Δ_0_, namely at the first dip, however, although the situation for *a*_2_ is just contrary, the currents carried by the two ARs are of the same direction and the magnitudes of *a*_1_ and *a*_2_ are slightly different. At the voltage value *V*_*g*(*c*)_, the critical Josephson currents *I*_*c*_ for the positive and negative directions are equal, which respectively correspond to the two different phase differences as detailed in Fig. 3, thus a 0 − *π* transition from the positive to negative direction for the *I*_*c*_ takes place. With the further increase of *V*_*g*_, *a*_1(2)_ becomes positive (negative) again at the second dip, then turns negative (positive) again at the third dip, where the former and latter are respectively corresponding to *π* − 0 and 0 − *π* transitions.

Physically, the 0 − *π* transition in SC/FM/SC junction is attributed to the tunneling Cooper pair acquiring a nonzero momentum due to FM exchange splitting in the FM region. However, in the present structure, compared with the case of no interedge coupling, since there is no difference between the spin-up and -down electrons, the 0 − *π* transition tuned by the gate voltage *V*_*g*_ should be just originated from the interedge coupling, which induces an additional *π* phase difference in *I*(*ϕ*). Specifically, in the context of the interedge coupling, the interedge backscattering between the edge states at two sides can occur at the interface between the left (right) and middle regions, but the intraedge backscattering is still prevented on account of the helical nature of the edge states^7–9^. The wave from the AR at interface *x* = 0 in edge 1 and that from the interedge backscattering at the interface in edge 2 are destructively coherent, leading to the additional *π* phase difference and the resultant 0 − *π* transition modulated by *V*_*g*_. The additional *π* phase difference can be also seen from the two ARs *a*_1(2)_ varying with *V*_*g*_ under the interedge coupling, as shown in Fig. 2(b). The physical mechanism of 0 − *π* transition by *V*_*g*_ stemming from the interedge coupling can be described as follows. With increasing *V*_*g*_, the mismatch from the quasiparticle wave in the middle region, which can be effectively regarded as a barrier at the interface, gets large. It follows that the AR is suppressed, whereas the normal reflection is stimulated. Particularly, due to the interedge coupling, the fraction of the former in edge 2 is strongly inhibited and that of the latter is largely enhanced. As their competition reaches to a certain degree, the direction of the current in edge 2 is reversed. As a result, the supercurrent in edge 1 also exhibits sign reversal with it, thus the AR *a*_1(2)_ jumps from the positive (negative) to negative (positive) value and a dip of *I*_*c*_ with *V*_*g*_ emerges, resulting in a 0 − *π* transition. The same mechanism is for the subsequent *π* − 0 and 0 − *π* transitions. Furthermore, the 0 − *π* transition tuned by *V*_*g*_ in turn can demonstrate the helical spin texture of the edge states.

Next, the characteristic variations of the highly nonsinusoidal current-phase relation *I*(*ϕ*) in the vicinity of the crossover between 0 and *π* states, for instance, corresponding to the first dip of red dashed line in Fig. 2(a), is plotted at different *V*_*g*_ with *α*_2_ = 0.7Δ_0_ in Fig. 3(a). It is seen that with the enhancement of *V*_*g*_, the junction will evolve from the normal 0 state (*V*_*g*_ = 4.2Δ_0_) to the abnormal *π* one (*V*_*g*_ = 16Δ_0_) with the magnitude of the gate voltage of the corresponding dip *V*_*g*(*c*)_ = 11Δ_0_. The lines in Fig. 3(a) typically show that the critical Josephson current *I*_*c*_ could be reversed with suitable parameters, and a *π* state could form in the system. *I*_*c*_ for the 0 state corresponds to *ϕ* being around *π*, while that for the *π* state is at *ϕ* = 0, which is different from the situation for the conventional 0 − *π* transition. At the crossover point, *I*_*c*_ for the 0 state is equal to that for the *π* state, as has been mentioned above. Tracking the absolute value of the current with increasing *V*_*g*_ from Fig. 3(a), one finds that *I*_*c*_ never becomes zero and has a large residual value at the 0 − *π* transition point. In addition, the region of coexisting 0 and *π* states is considerably large, which can be employed, e.g., in the design of a *π* SQUID with improved accuracy, a typical device with an effectively two times smaller flux quantum^19, 20^. To show more clearly that the 0-and *π*-states are just the ones whose corresponding *ϕ* are respectively 0 and *π*, we present the *ϕ* dependences of the free energy *F* in the junction at the crossover point (*V*_*g*(*c*)_ = 11Δ_0_), 0-state (*V*_*g*_ = 4.2Δ_0_), and *π*-state (*V*_*g*_ = 16Δ_0_), as illustrated by Fig. 3(b). It is demonstrated the minimum of the *F* in the two states is actually located at *ϕ* = 0 and *ϕ* = *π*, respectively, not only the crossover point, indicating that the junction really owns the 0- and *π*-states as in usual metallic SC/FM/SC junctions of refs 11–13, and thus can be possibly applied to a qubit or SQUID. The relation between the *I*(*ϕ*) and *F* is of slight novelty and a little different from that of usual situation, just originating from the interedge coupling inducing the 0 − *π* transition.

### Experimental feasibility

We now discuss the feasibility of experiments using the QSH insulator in HgTe/CdTe quantum wells^36^. It was reported that with the separation between edge channels equaling to 400 nm^36^, the interedge coupling strength *α*_2_ was estimated to be about 10 *μ*eV^8^. This interedge coupling strength is of the same order as the proximity-induced superconducting gap in the edge states with the maximum vaule of Δ_0_ estimated by 20 *μ*eV for Ti/Al superconducting material^36^, meaning that the interedge coupling strength in our calculation is experimentally achievable. Furthermore, the bulk band gap of the HgTe/CdTe quantum well is typically *E*_*g*_ ~ 1 − 30 meV^3, 4^ and the gate voltage *V*_*g*_ in our calculation is from 0 to 50Δ_0_, i.e., *V*_*g*_ ≤ 1 meV, therefore, the requirement of the transport at the Fermi energy inside the bulk gap can also be satisfied. The thermal activation *k*_*B*_ *T* ≈ 0.057Δ_0_ at the fixed temperature *T* = 0.1*T*_*c*_, reaching a maximum of 1.14 *μ*eV, is much less than the bulk band gap as well. The gate voltage of around 10Δ_0_ ~ 0.2 meV can be acceptable for qubit or SQUID application, which is addressed in what follows. Although the present *π* junction in this work can be considered metallic, being a little similar to the *π* SC/FM/SC junction of ref. 18, it is characterized by gapless helical edge states and is quasi one-dimensional for the flow of the supercurrent. The expected heat generation by *V*_*g*_ ~ 0.2 meV is estimated to be 0.04Δ_0_, being smaller than the thermal activation *k*_*B*_ *T*, are distributed on edges 1 and 2, therefore the consequent effect can be greatly reduced, especially after long decoherence time or even by applying a diluton refrigerator as usual in ref. 37. Also, the expected heat generation by *V*_*g*_ is found to be tuned by the interedge coupling. It is demonstrated from our calculation that the Andreev bound state energy in the present *π* junction is always smaller than 0.5Δ_0_, but is not too small. And hence, if the present *π* junction and a 0 junction constitute a superconducting qubit as in ref. 18, the present metallic *π* junction is also well gapped in energy gap of two-level quantum state for the qubit where the quantumn tunneling occurs. This implies that the quasiparticle tunneling which can cause the dissipation is also strongly suppressed at low temperatures. Though the heat generation may be of influence on the two-level quantum state of the qubit, thus leading to the slight shortening of decoherence time, it is a lot less than the energy gap between the two-level quantum states, which can be the same order in magnitude as that in ref. 18. In other words, the heat generation would be strongly overwhelmed by the energy gap, meaning no significant effect on the decoherence time. In particular, if manipulating the interedge coupling appropriately, one can not only prolong the decoherence time adequately but also reduce the heat generation fully.

In summary, we have proposed one setup for Josephson effect based on a narrow QSH insulator strip, which is shown to reveal an obvious 0 − *π* transition tuned by gate voltage due to the interedge backscattering. It is also demonstrated that the setup has three advantages: (1) the helical particles travel in the QSH insulator edge dissipationlessly due to the helicity conservation of the carriers, (2) the critical Josephson current *I*_*c*_ at the 0 − *π* transition point has a considerable residual value, and (3) the region of coexisting 0 and *π* states is found fairly large. The setup can be therefore used to design the low-energy dissipation supercurrent switch with high efficiency and a *π* SQUID with improved accuracy. The devices are likely to be achieved in mercury quantum wells and also provide an evidence of the helical spin texture of the edge states in the QSH insulator.
